# Forty Years of Stress & Coping Research at the VA Normative Aging Study

**DOI:** 10.1093/geroni/igaf122.1458

**Published:** 2025-12-31

**Authors:** Carolyn Aldwin

**Affiliations:** Oregon State University, Corvallis, Oregon, United States

## Abstract

The VA Normative Aging Study (NAS) began collecting data in the 1980s on trauma, stressful life events (SLEs), hassles, and coping. At that time, the idea that psychosocial stress affected health was controversial, with critics arguing that stress was simply confounded with personality. Our first study showed that neuroticism did predict stress 10 years later, but both SLEs and hassles had independent effects on mental health. Further, combat exposure (CE) had long-term effects on mental health. WWII Veterans with moderate CE were 13 times more likely to experience PTSD symptoms 45 years later, especially among those who reported negative appraisals of their military service, poor social support and negative homecoming experiences. While CE did not predict mortality, those who also experienced civilian trauma were 16% more likely to die prematurely. However, CE also had positive effects on well-being in late life, especially for those with positive appraisals of their military service. While SLEs and hassles generally declined with age, there were marked individual differences in patterns of stress across the lifespan; those with consistently elevated SLEs had a 42% greater risk for premature mortality, while those with consistently strong reactions to hassles had a 63% greater risk. Coping strategies also decreased with age, but coping efficacy was usually maintained, suggesting more efficient coping in late life. Those with elevated coping effort had a 14% greater risk of premature mortality. Thus, NAS research showed conclusively that stress and coping processes had marked effects on health in later life, independent of personality.

